# Lamotrigine versus inert placebo in the treatment of borderline personality disorder: study protocol for a randomized controlled trial and economic evaluation

**DOI:** 10.1186/s13063-015-0823-x

**Published:** 2015-07-18

**Authors:** Mike J. Crawford, Rahil Sanatinia, Barbara Barrett, Sarah Byford, Gillian Cunningham, Kavi Gakhal, Geof Lawrence-Smith, Verity Leeson, Fenella Lemonsky, Georgia Lykomitrou, Alan Montgomery, Richard Morriss, Carol Paton, Wei Tan, Peter Tyrer, Joseph G. Reilly

**Affiliations:** Centre for Mental Health, Imperial College London, Du Cane Road, London, W12 ONN UK; King’s Health Economics, King’s College London, De Crespigny Park, London, SE5 8AF UK; School of Medicine, Pharmacy and Health, Durham University, Stockton Road, Durham, DH1 3LE UK; Oxleas NHS Foundation Trust, Pinewood Place, Dartford, DA2 7WG UK; Faculty of Medicine & Health Sciences, University of Nottingham, Queen’s Medical Centre, Nottingham, NG7 2UH UK

**Keywords:** Borderline personality disorder, Mood stabilizer, Lamotrigine, Randomized trial

## Abstract

**Background:**

People with borderline personality disorder (BPD) experience rapid and distressing changes in mood, poor social functioning and have high rates of suicidal behaviour. Several small scale studies suggest that mood stabilizers may produce short-term reductions in symptoms of BPD, but have not been large enough to fully examine clinical and cost-effectiveness.

**Methods/Design:**

A two parallel-arm, placebo controlled randomized trial of usual care plus either lamotrigine or an inert placebo for people aged over 18 who are using mental health services and meet diagnostic criteria for BPD. We will exclude people with comorbid bipolar affective disorder or psychosis, those already taking a mood stabilizer, those who speak insufficient English to complete the baseline assessment and women who are pregnant or contemplating becoming pregnant. Those meeting inclusion criteria and provide written informed consent will be randomized to up to 200mg of lamotrigine per day or an inert placebo (up to 400mg if taking combined oral contraceptives).Participants will be randomized via a remote web-based system using permuted stacked blocks stratified by study centre, severity of personality disorder, and level of bipolarity.

Follow-up assessments will be conducted by masked researchers 12, 24 weeks, and 52 weeks after randomization. The primary outcome is the Zanarini Rating Scale for Borderline Personality Disorder (ZAN-BPD). The secondary outcomes are depressive symptoms, deliberate self-harm, social functioning, health-related quality of life, resource use and costs, side effects of treatment, adverse events and withdrawal of trial medication due to adverse effects.

The main analyses will use intention to treat without imputation of missing data. The economic evaluation will take an NHS/Personal Social Services perspective. A cost-utility analysis will compare differences in total costs and differences in quality of life using QALYs derived from the EQ-5D.

**Discussion:**

The evidence base for the use of pharmacological treatments for people with borderline personality disorder is poor. In this trial we will examine the clinical and cost-effectiveness of lamotrigine to assess what if any impact offering this has on peoples’ mental health, social functioning, and use of other medication and other resources.

**Trial registration:**

Current Controlled Trials ISRCTN90916365 (registered 01/08/2012)

## Background

People with borderline personality disorder (BPD) have disturbances in mood which include affective instability, outbursts of anger, and feelings of emptiness. These are accompanied by negative thoughts about self and poor interpersonal functioning [1]. As many as 2 % of people have BPD [2], and levels are far higher among those in contact with mental health services, particularly inpatient units, where up to a fifth of people may have this diagnosis [3]. People with BPD have poor social functioning, are socially isolated, and are usually unemployed or on long-term sick leave [4]. They also have high rates of deliberate self-harm and a rate of completed suicide that is 50 times that in the general population [5].

No drugs are currently licensed for BPD; however, people with this condition are usually prescribed large amounts of psychotropic medication [6]. The prominence of affective instability among people with BPD has led to interest in the role that mood stabilizers might play in helping people with this condition. Concerns about toxicity in overdose and harm to children born to women taking these drugs have limited research to date. Evidence from cohorts of people receiving treatment for epilepsy suggests that valproate and lamotrigine may be safer in overdose than carbamazepine [7] and that congenital malformations are more common among people taking valproate than among those taking lamotrigine [8].

To date, there have been two small-scale randomized trials of lamotrigine. The first involved 24 women recruited mainly from advertisements placed in primary care practices. In comparison with people taking placebo, those taking up to 200 mg of lamotrigine daily were found to have lower levels of anger 8 weeks later [9]. The second trial recruited 28 men and women through websites and television and radio advertisements. Those randomly assigned to receive up to 225 mg of lamotrigine were found to have lower levels of affective instability and impulsiveness 12 weeks later [10]. These studies have a number of limitations, including their focus on short-term outcomes, small sample sizes, and the absence of an economic evaluation. Furthermore, people who took part in them may not have had the degree of severity of the disorder that is seen among people who are generally receiving treatment in secondary care mental health services. Although these studies have demonstrated the feasibility of randomized trials for people with BPD, they have not been sufficiently large to fully test the clinical effectiveness and cost-effectiveness of this approach to trying to help people with this condition.

### Research objectives

The main objectives of the study are to find out whether adding lamotrigine to usual care for people with BPD, compared with those prescribed an inert placebo, improves mental health, social functioning, and quality of life and whether it reduces the incidence of suicidal behaviour and lowers the amount of anti-psychotic and other psychotropic medication that people are prescribed. We will also examine whether adding lamotrigine to usual care for people with BPD, compared with those prescribed an inert placebo, is associated with a higher incidence of side effects and whether adding lamotrigine provides a cost-effective use of resources.

## Methods/Design

**Fig. 1:**
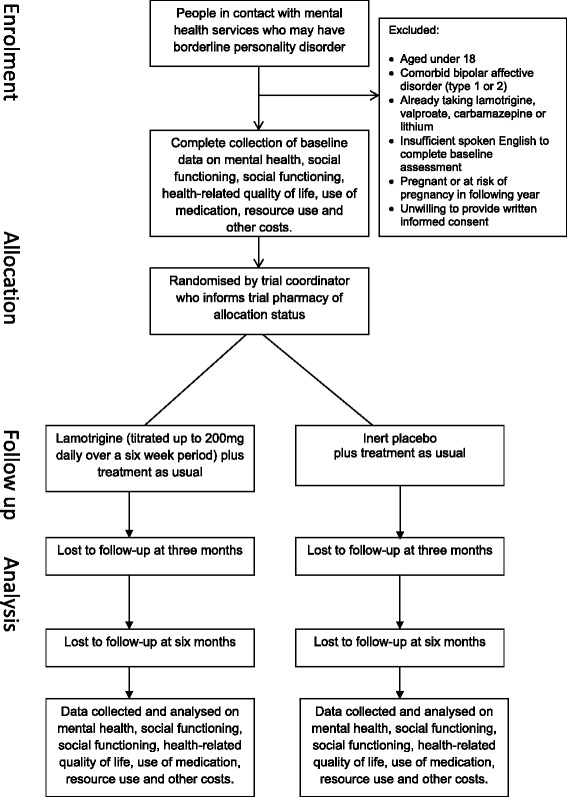
Study flow chart

The LABILE trial (Lamotrigine And Borderline personality disorder: Investigating Long-term Effectiveness trial) is a multi-centre, two-arm, parallel group, double-blind, placebo-controlled randomized trial with 3-, 6-, and 12-month follow-up assessment. The trial includes an integrated clinical and economic evaluation. A summary of trial design is presented in Fig. 1.

### Study setting

Study participants will be recruited from secondary care mental health services in England, including inpatient units, outpatient clinics, and community mental health teams. There are six recruitment centres in London (Central and North West London NHS Foundation Trust, Oxleas NHS Foundation Trust, and West London Mental Health NHS Trust), the East Midlands (Nottinghamshire Healthcare NHS Foundation Trust and Derbyshire Healthcare NHS Foundation Trust), and the North East of England (Tees, Esk and Wear Valleys Foundation NHS Trust).

### Eligibility criteria

The target population is adults who are at least 18 years old and who meet DSM-IV (Diagnostic and Statistical Manual of Mental Disorders, 4th Edition) diagnostic criteria for BPD [1] and are willing and able to provide written informed consent to take part in the study. Eligibility will be assessed by trained researchers. Exclusion criteria are the following:

i). current or past comorbid bipolar affective disorder (type I and II) or psychotic disorder (schizophrenia, schizoaffective disorder, or mood disorder with psychotic features),
ii). currently receiving a mood stabilizer (lithium, carbamazepine, or valproate) or have done so within the previous four weeks,
iii). known medical history of liver or kidney impairment,
iv). cognitive or language difficulties that would preclude people from providing informed consent, and
v). any woman who is pregnant or planning a pregnancy or any woman of child-bearing age unless using adequate contraception.

### Interventions

Those in the active arm of the trial will receive encapsulated generic lamotrigine titrated according to the established British National Formulary protocol (http://www.bnf.org/bnf/index.htm) but with the titration occurring at standardised 14-day intervals. The dose will be altered if participants are also taking the combined oral contraceptive pill, which affects the metabolism of lamotrigine. For those not taking the combined oral contraceptive pill, the starting dose will be 25 mg per day. Depending on the response and tolerance, it will be increased to 50 mg after 2 weeks, 100 mg after 4 weeks, and 200 mg per day after 6 weeks and thereafter. For those who are taking the combined oral contraceptive pill, the starting dose will be 25 mg per day. This will be increased to 50 mg after 2 weeks, 100 mg after 4 weeks, 200 mg after 6 weeks, 300 mg after 8 weeks, and 400 mg per day after 10 weeks.

Trial medication will be issued to patients fortnightly to cover the dose titration period, and subsequent 4-weekly packs will be issued once the maintenance dose is reached. This will ensure that non-adherence and subsequent study withdrawal are dealt with promptly and that large supplies of medication cannot be accumulated by patients who may be at risk of overdosing. Supplies will be issued by the relevant hospital pharmacy services. The lamotrigine and placebo capsules will be kept in a suitable temperature-controlled environment at each site.

Those in the placebo arm of the trial will receive capsules, identical in appearance to those containing active lamotrigine, but backfilled instead with lactose monohydrate. This will be prescribed in the same regime as that used in the active arm of the trial.

Usual care will comprise contact with primary and secondary health services, including access to psychological treatment services and inpatient admission if required. No restrictions will be imposed on the use of other treatments, except that those who remain in the trial will not be prescribed lamotrigine (aside from trial medication) or another anti-epileptic mood stabilizer (lithium, carbamazepine, or sodium valproate). Therefore, our approach will be primarily to record the use of all other medication, document details of dosage, and ensure the follow-up of all randomly assigned participants, irrespectively of the medication they subsequently receive.

In accordance with the current revision of the Declaration of Helsinki (amended October 2000, with additional footnotes added 2002 and 2004), a participant will have the right to stop trial treatment and to withdraw from the trial at any time and for any reason, without prejudice to his or her future medical care by the physician or at the institution, and is not obliged to give his or her reasons for doing so. The investigator may withdraw a participant from trial treatment at any time in the interests of the participant’s health and well-being or for administrative reasons. A trial participant will be withdrawn from taking the trial medication if he or she experiences a rash that is clinically judged as being associated with lamotrigine. The date and reason for termination of treatment will be recorded. Trial follow-ups will continue after treatment has been withdrawn, unless the participant withdraws consent.

### Assessments

The timing and sequence of all assessments are summarised in Table 1*.*

**Table 1:** Study assessment schedule

Assessments	Screening	Baseline	12-week follow-up	24-week follow-up	52-week follow-up
Structured Clinical Interview for DSM-IV Axis II Personality disorders	X	-	-	-	-
Structured Clinical Interview for DSM-IV (SCID-I)^a^	X	-	-	-	-
Hypomanic Checklist	X	-	-	-	-
Alcohol, Smoking and Substance Involvement Screening Test (ASSIST)	X	-	-	-	X
Morisky scale	-	-	X	X	X
Zanarini Rating Scale for Borderline Personality Disorder (ZAN-BPD)	-	X	X	X	X
Beck Depression Inventory	-	X	X	X	X
Acts of Deliberate Self Harm	-	X	X	X	X
Social Functioning Scale	-	X	X	X	X
EuroQoL-5D	-	X	X	X	X
Side Effect Scale	-	X	X	X	X
Modified Adult Service User Schedule	-	X	X	X	X

*DSM-IV* Diagnostic and Statistical Manual of Mental Disorders (4th edition), *EuroQoL-5D* European quality of Life-5 dimensions

^a^Section on bipolar affective disorder (type I and II)

#### Baseline

At baseline, we will assess eligibility by using the Structured Clinical Interview for Axis II Personality Disorders (SCID-II) [11]. The SCID-II provides a reliable assessment of BPD [12], has a shorter administration time than other semi-structured interviews used to assess BPD, and can be completed within 1 h. We will use data from the SCID-II to establish the severity of the participant’s personality disorder [13]. We will use the Structured Clinical Interview for Axis I Disorders (SCID-I) [14] to assess whether potential participants have bipolar affective disorder (type I and II) and to exclude those who do. Hypomanic symptoms will be assessed by using the Hypomanic Checklist [15], a relatively short screening questionnaire that can distinguish those with bipolar disorder from those with unipolar depression.

#### Primary outcome

The primary outcome is symptoms of BPD measured by using the Zanarini Rating Scale for Borderline Personality Disorder (ZAN-BPD) [16] 52 weeks after randomization. The ZAN-BPD is a widely used measure of symptoms and behavioural problems experienced by people with BPD. It includes measures of anger, impulsivity, and affective instability. The ZAN-BPD has been used in previous studies of pharmacological and psychological treatments for people with BPD [10, 17, 18]. It is reliable (intraclass correlation coefficients for inter-rater reliability of 0.96 and test-retest reliability of 0.93), has high convergent validity with structured clinical ratings of symptoms of BPD (e.g., Revised Diagnostic Interview for Borderlines), and is sensitive to change [16].

#### Secondary outcomes

1. Scores on the ZAN-BPD in the 52 weeks after randomization using repeated measures analysis of data collected at 12-, 26-, and 52-week follow-up.
2. Total score on the 21-item Beck Depression Inventory [19] at 12, 24, and 52 weeks. The Beck Depression Inventory has been widely used as a self-completed questionnaire, provides a valid assessment of the severity of depressive symptoms, and can be completed in less than 10 min [20].
3. Incidence and severity of suicidal behaviour, assessed by using the Acts of Deliberate Self-Harm Inventory [21] at 12, 24, and 52 weeks. This structured interview collects detailed information about the number and severity of episodes of self-harm and has been used successfully in other trials of treatments for people with BPD [22].
4. Social functioning, assessed by using the Social Functioning Questionnaire at 12, 24, and 52 weeks. This questionnaire is an eight-item self-report scale that asks people about problems across a range of settings that people with personality disorder often experience [23].
5. Health-related quality of life, assessed by using the EuroQoL-5D (European quality of Life-5 dimensions) [24] at 12, 24, and 52 weeks. The EuroQOL-5D provides a brief and reliable measure of health-related quality of life and is responsive to change in people with BPD [25].
6. Side effects, assessed at 12, 24, and 52 weeks by using a pro forma designed to cover the possible side effects listed in the British National Formulary entry for lamotrigine [26].
7. Use of alcohol and other drugs at 52 weeks, assessed by using the Alcohol, Smoking and Substance Involvement Screening Test (ASSIST) [27]. This short questionnaire provides a reliable and valid screening test for problem substance use [28].
8. Use of concomitant anti-psychotic and other psychotropic medication defined as the proportion of people taking anti-psychotics, anti-depressants, benzodiazepines, and hypnotics 24 and 52 weeks after randomization.
9. Total cost of health and social services; resource use information will be collected at 12, 24, and 52 weeks by using the Adult Service Use Schedule adapted for use in this trial on the basis of previous research involving people with personality disorders [29]. This questionnaire collects detailed data on the use of all hospital and community health and social care services.

All assessments will be conducted by researchers who have been trained to use study instruments by clinicians with expertise in the assessment of people with personality disorder.

#### Adherence

Adherence with study medication will be assessed at 12-, 24-, and 52-week assessments by using the Morisky Scale [30]. This is a four-item questionnaire which provides a valid estimate of adherence with psychotropic medication. In addition, researchers will ask participants about their use of medication when each prescription is renewed and record any treatment breaks.

### Sample size

The sample size calculation for the study is based on our primary hypotheses that, for people with BPD who are in contact with mental health services, the addition of lamotrigine to their usual treatment will reduce symptoms of their disorder at 52-week follow-up, according to the ZAN-BPD. The ZAN-BPD has been used to examine the clinical effectiveness of a range of psychological and pharmacological treatments for people with BPD. In a trial of problem-solving therapy compared with treatment as usual, Blum and colleagues [17] found improvements in mental health and reduced use of emergency medical services among those who were randomly assigned to problem-solving therapy. This was associated with a difference of 3.6 (standard deviation (SD) = 6.9) in total ZAN-BPD score.

The ZAN-BPD rating scale was also used to examine the clinical effectiveness of lamotrigine for people with BPD in a randomized trial conducted by Reich and colleagues [10]. In this small trial (*n* = 28), a non-statistically significant difference in total score (5.6, SD = 6.75) on the ZAN-BPD was found at 12 weeks. Seventeen people (61 %) in the trial completed all 12 weeks of the study, and levels of adherence to trial medications in those who completed the study were judged to be high.

It is anticipated that levels of adherence to trial medications may be lower than in the study by Reich and colleagues, and the study has been powered on the basis of a minimal clinically significant difference in ZAN-BPD score of 3.0 (SD = 6.75). The sample size was calculated by using Stata version 13 (StataCorp LP, College Station, TX, USA).

Two hundred fourteen participants (107 receiving lamotrigine and 107 receiving placebo) would need to be randomly assigned to have 90 % power to detect a minimal clinically relevant difference of 3.0 (SD = 6.75) in total score on the ZAN-BPD at 52 weeks by using a 0.05 two-sided level of statistical significance. To take account of 15 % loss to follow-up at 52 weeks, sample size has been increased to 252.

### Assignment of interventions

After consenting to participation and completing screening assessments, patients who are found to be eligible will be randomly allocated to the intervention or comparator arm of the trial by the automated randomization service operated by Nottingham Clinical Trials Unit. Equal numbers of participants will be randomly assigned to each arm of the trial. Stratification will occur by study centre, severity of personality disorder (simple or complex personality disorder according to criteria developed by Tyrer and Johnson [13]), and extent of bipolarity (using a score of more or less than 14 on the Hypomania Checklist [15]).

### Blinding

The randomization service will generate a unique trial identification number for that participant, which will be used on the case report forms (CRFs). Blinding of investigators, researchers, clinicians, and patients will be maintained until all data entry and processing are complete and the database has been locked. All patients, carers, and study personnel will be blinded to treatment assignment. The study statistician will also be blind to trial arm allocation. Premature disclosure of allocation runs the risk of introducing bias and invalidating the trial results. Therefore, masking of treatment allocation will be maintained during the course of the trial unless the following occur: a serious adverse event arises that clinically requires disclosure, a trial drug overdose that requires disclosure occurs, or the participant becomes pregnant.

In anticipation of an emergency, investigators, clinicians, and participants will be provided with the telephone number for a 24-h emergency unblinding service at the Medical Toxicology and Information Services, which offers medical support. This system will allow a medical request for unblinding in the event of a medical emergency to be responded to 24 h a day, 7 days a week. Procedures will be put in place to verify the identity of the participant and caller, and the decision on whether to reveal the study medication allocation will be based on a set of criteria for judging clinical need. All requests for unblinding will be recorded.

After the completion of the 52-week follow-up assessment and regardless of whether they withdraw from the study early or complete the participation period in full, an email will be sent to the referring psychiatrist informing them of the participant’s trial arm allocation. Where a participant has completed the participation period in full, this will allow the prescriber time to make arrangements for the participant to continue on lamotrigine if appropriate and desired. Upon completion of their 52-week follow-up assessment, the participant will be advised to contact their psychiatrist to discuss their trial arm allocation if he or she wishes to know whether they were taking the active or placebo medication.

### Study logistics

#### Recruitment

Potential participants will initially be approached about the study by any health-care professional who is involved in their care provided that the consultant psychiatrist for the team has agreed in principle that patients under their care may take part in the study.

If a psychiatrist or other health-care professional has a patient under their care who they believe meets the eligibility criteria for the study, they will introduce the patient to the study at an appropriate time by briefly describing it and provide an information sheet. The information sheet will include an explanation of the exact nature of the trial, the requirements of the protocol, any known adverse effects of the trial medicine, and any known risks involved in taking part. It will be clearly stated that the patient is free to withdraw from the trial at any time for any reason without prejudice to future care and with no obligation to give the reason for withdrawal.

The patient must provide verbal agreement to discuss their eligibility and possible enrolment into the trial with a member of the research team before any further step in the study process can take place. If a patient does not give verbal agreement to discuss their eligibility and possible enrolment into the trial, no further aspect of the screening process will be carried out at that time. However, where a patient later decides that they are willing to be considered for entry into the trial, previous refusal does not preclude this. Where verbal agreement is given, the patient will be assigned a screening number and contacted by the research team to discuss consent.

Potential participants will be given no less than 24 h from receiving the information sheet to consider the information and the opportunity to question the investigator, their general practitioner (GP), or other independent parties regarding participation in the trial. Written informed consent then will be obtained and will include permission for the LABILE research team to notify the patient’s GP and consultant (who may be the referring psychiatrist) about the enrolment of their patient into the trial. Additionally, the patient will be asked whether a family member or friend can be contacted solely for the purpose of helping the team to obtain follow-up data, for which a separate written informed consent will be required. The patient will not be excluded from the study if he or she does not give consent for the research team to contact family members or friends. A copy of the signed informed consent form(s) will be given to the patient and their consultant. The original signed form(s) will be retained at the trial site.

#### Screening and baseline

If consent is given and documented, a screening assessment CRF will be completed with the participant. If the participant fulfils the eligibility criteria, they will complete the baseline assessment and be randomly assigned. After randomization, the participant’s GP and consultant will be informed of their enrolment into the trial.

#### Follow-up

The 12-, 24-, and 52-week assessments (Table 1) will be scheduled to coincide with the supplying of the participant’s study medication. Prior to preparing each new prescription, the psychiatrist or researcher will contact the participant to elicit details of any adverse events and to ascertain whether they wish to continue with the trial. If the participant continues to experience adverse event(s), further monitoring will be performed even when they are no longer being prescribed the study medication. Further follow-up by visit or telephone call will be arranged as required. All participants will be offered a £20 honoraria after completion of the 52-week follow-up interview.

### Data management and analysis

Data will be entered onto a secure web-based data entry system. Access will be restricted by user identifiers and passwords (encrypted by using a one-way encryption method). The database will be backed up daily in encrypted form, and offsite copies will be made at regular intervals. Study data will be archived securely and then safely destroyed after 15 years. Analysis and reporting of the trial will be in accordance with CONSORT (Consolidated Standards of Reporting Trials) guidelines. Further details about the statistical analyses will be provided in the statistical analysis plan which will be finalised prior to completion of data collection, database lock, and unblinding of the study.

Continuous variables will be summarised in terms of the mean, SD, median, lower and upper quartiles, minimum, maximum, and number of observations. Categorical variables will be summarised in terms of frequency counts and percentages. Descriptive statistics of demographic and clinical measures will be used to examine balance between the randomized arms at baseline. The primary approach to between-group comparisons will be to analyse participants according to the group to which they were allocated, regardless of treatment received, and without imputation of missing outcome data. All data will be analysed by using Stata version 13 or a later version.

Appropriate descriptive statistics for ZAN-BPD score will be reported for each randomly assigned group at each scheduled follow-up time point. There are two proposed analyses of ZAN-BPD data. For our primary analysis, ZAN-BPD score at 52-week follow-up, randomly assigned groups will be compared by using a generalised linear model for continuous outcome adjusted by baseline ZAN-BPD score, centre, severity of personality disorder (simple or complex), and the extent of bipolarity (score of at least 14 or of less than 14). The effectiveness parameter comparing lamotrigine plus usual care with usual care alone will be the difference in mean ZAN-BPD score at 52 weeks along with 95 % confidence interval and exact *P* value.

For our main secondary analysis, ZAN-BPD scores at 12, 24, and 52 weeks will be compared by using a mixed model for repeated outcome measures adjusted by the same stratification variables used for the primary analysis. We will investigate whether any treatment effects were sustained or emerged later by including an interaction term between treatment group and time in the model. In the absence of a time effect, the effectiveness parameter will be the average difference in mean ZAN-BPD score over the 52-week period along with 95 % confidence interval and exact *P* value.

Sensitivity analyses will be conducted for both analyses (i) to further adjust for any variable with marked imbalance at baseline, (ii) to investigate the impact of missing data by using multiple imputation, and (iii) to investigate the effect of treatment adherence.

Analysis of secondary outcomes will follow a similar approach by using appropriate mixed effects regression models (for example, logistic for binary outcomes) (that is, comparison at both 52-week follow-up and by using repeated measures analysis of follow-up data collected at 12, 26, and 52 weeks).

#### Health economic analysis

The economic evaluation will take the NHS/Personal Social Services perspective preferred by the National Institute for Health and Clinical Excellence [31] and shown to be the key cost sector in previous research among people with BPD [29]. Data on the use of health and social services will be collected by using a modified version of the Adult Service Use Schedule adapted for use in this population on the basis of previous research in this area [29]. The cost of lamotrigine will be calculated by using the generic cost listed in the British National Formulary and the cost of the time with the dispensing clinician by using national UK unit costs. National UK unit costs will be applied to medication, hospital contacts, and community health and social services [32, 33].

Differences in mean costs will be analysed by using standard parametric *t* tests with the validity of results confirmed by using bias-corrected, non-parametric bootstrapping (repeat re-sampling) [34]. Despite the skewed nature of cost data, this approach is recommended to enable inferences to be made about the arithmetic mean [35]. Cost-effectiveness will be assessed through the calculation of incremental cost-effectiveness ratios [36] and will be explored in terms of cost utility by using quality-adjusted life-years derived from the EuroQoL-5D and cost-effectiveness by using the ZAN-PD. Uncertainty around the cost and effectiveness estimates will be represented by cost-effectiveness acceptability curves [37]. All analyses of cost will be adjusted for baseline stratification variables and baseline costs.

### Data monitoring

The project will be overseen by a trial steering committee (TSC) made up of a representative of the project funder and independent representatives of service users and providers. An Independent Data Monitoring and Ethics Committee will monitor recruitment of study participants, ethical issues of consent, quality of data (including missing data), the incidence of adverse events, and any other factors that might compromise the progress and satisfactory completion of the trial. The group will have access to unblinded data if requested. It will be chaired by an independent academic and report to the TSC.

Day-to-day running of the project will be overseen by a trial management group. The role of the group will be to monitor all aspects of the conduct and progress of the trial, ensure that the protocol is adhered to, and take appropriate action to safeguard participants and the quality of the trial itself. The group will act in accordance with decisions made by the TSC. People with experience using services will be represented on the TSC and contribute to the design, conduct, and reporting of study findings.

### Ethics

Approval for the research was given by the London Central Research Ethics Committee (reference number 12/LO/1514) and by the Research and Development departments of the participating NHS Trusts. We will ensure that the trial is conducted in full conformity with the current revision of the Declaration of Helsinki (last amended October 2000, with additional footnotes added 2002 and 2004) and with the Medicines for Human Use (Clinical Trials) Regulation 2004 transposed into law from the EU Clinical Trials Directive 2001/20/EC, the EU Good Clinical Practice Directive 2005, and all current and future acts and requirements pertaining to its conduct.

Each study participant will be assigned a unique trial identification number at the start of the assessment process. This number will be written on all clinical assessment forms/datasheets and databases used to record data on study participants. A hard copy of a record sheet linking patient identity, contact details, and trial identification number for all participants will be kept at each site. It will be placed in the investigator site file, in a locked filing cabinet, separate from the paper CRFs and other documents relating to a participant, which will be anonymised. Recorded data will be entered onto an electronic data management system that will use the trial identification number rather than the participant’s name or other information that could identify them.

## Discussion

The LABILE trial will be the first study to examine the long-term clinical effectiveness and cost-effectiveness of lamotrigine for people with BPD. As such, the trial has the potential to help guide prescribers and patients when deciding whether this drug could help reduce the distress they experience and improve their quality of life.

Current guidance from the National Institute for Health and Care Excellence states that drug treatments should “not be used for BPD or for the individual symptoms or behaviour associated with the disorder” [38]. This position was endorsed by subsequent guidance issued by the National Health and Medical Research Council in Australia [39] but contrasts with earlier American guidelines which state that mood stabilizers should be considered as a second-line treatment for affective dysregulation in patients with BPD [40]. The need for further trials of pharmacological treatments for people with borderline personality was highlighted in a Cochrane systematic review in 2008 [41]. Previous reports have highlighted the uncertainty that prescribers currently experience when considering whether to use pharmacological approaches to help people with BPD [42], and we believe that this trial can help reduce some of this uncertainty.

Strengths of the LABILE study are that it has a clear primary hypothesis based on an outcome measure which assesses core features of BPD. As BPD is a long-term condition and previous placebo-controlled trials have examined outcomes only over 8 to 12 weeks, we want to examine the longer-term effects of offering people this treatment. For this reason, our primary outcome measure is score on the ZAN-BPD at 52 weeks. However, BPD is also a fluctuating condition, and it is possible that patients may derive some benefit from treatment even if these are not seen at 52 weeks. For this reason, the first of our secondary outcomes is symptoms of BPD over the 52 weeks after randomization using repeated measures analysis of data collected at 12-, 26-, and 52-week follow-up. This measure will allow us to examine whether patients who are offered lamotrigine have improved mental health in the year after they are first offered this treatment.

Other strengths are that we are recruiting participants from a broad range of secondary care mental health services and we have limited our exclusion criteria with the aim of generating findings that are generalisable across the NHS in England and in other countries with similar health-care systems. Finally, the study is collecting detailed information about resource use and other costs that will enable us to conduct a high-quality economic evaluation of the impact of adding lamotrigine to usual care of people with BPD.

This trial faces a number of challenges which will need to be overcome if we are to recruit to target. In addition to pressures of workload and other factors that may deter clinicians from referring people to clinical trials, clinicians may be concerned about the impact of the trial on what can sometimes be strained relationships with patients. National guidance on the treatment of people with BPD in England advises against the use of pharmacological treatments and this may deter some clinicians from referring people to the study. Potential participants may be concerned about being asked to take part in a study in which they might be allocated to an inert placebo for a 12-month period. In an attempt to overcome these obstacles, we have developed information for clinicians that emphasises the extent of off-label prescribing of psychotropic medication for people with personality disorder and reminds them that national guidance is based on the relative absence of evidence rather than evidence of lack of effect. Initial discussions with potential participants suggest that many recognise the limitations of current treatment options and that there is considerable interest in taking part in the study.

We have previously concluded that existing evidence is insufficient to recommend the use of lamotrigine or other mood stabilizers in clinical practice [42]. The LABILE study has the potential to reduce this uncertainty by generating high-quality evidence on the impact of adding this drug to the usual care that patients receive over the course of a 12-month period.

## Trial status

Recruitment is ongoing (218 participants recruited as of end of May 2015).

## Abbreviations

BPD: Borderline personality disorder
CRF: Case report form
EuroQoL-5D: European quality of Life-5 dimensions
GP: General practitioner
LABILE: Lamotrigine and borderline personality disorder: Investigating Long-term effectiveness
NHS: National Health Service
SCID-II: Structured Clinical Interview for Axis II Personality Disorders
SD: Standard deviation
TSC: Trial steering committee
ZAN-BPD: Zanarini rating scale for borderline personality disorder

## References

[1] American Psychiatric Association (1994). Diagnostic and Statistical Manual of Mental Disorders.

[2] Coid J, Yang M, Tyrer P, Roberts A, Ullrich S (2006). Prevalence and correlates of personality disorder in Great Britain. Br J Psychiatry.

[3] Hayward M, Slade M, Moran PA (2006). Personality disorders and unmet needs among psychiatric inpatients. Psychiatr Serv.

[4] Skodol AE, Gunderson JG, McGlashan TH, Dyck IR, Stout RL, Bender DS (2002). Functional impairment in patients with schizotypal, borderline, avoidant, or obsessive-compulsive personality disorder. Am J Psychiatry.

[5] Lieb K, Zanarini MC, Schmahl C, Linehan MM, Bohus M (2004). Borderline personality disorder. Lancet.

[6] Crawford MJ, Kakad S, Rendell C, Mansour NA, Crugel M, Liu KW (2011). Medication prescribed to people with personality disorder: the influence of patient factors and treatment setting. Acta Psychiatr Scand.

[7] Sukumaran S, Herbert J, Tracey J, Delanty N (2005). Safety of newer generation anti-epileptic drugs in non-accidental overdose: an Irish population study. Seizure.

[8] Harden CL (2008). Antiepileptic drug teratogenesis: what are the risks for congenital malformations and adverse cognitive outcomes?. Int Rev Neurobiol.

[9] Tritt K, Nickel C, Lahmann C, Leiberich PK, Rother WK, Loew TH (2005). Lamotrigine treatment of aggression in female borderline-patients: a randomized, double-blind, placebo-controlled study. J Psychopharmacol.

[10] Reich DB, Zanarini MC, Bieri KA (2009). A preliminary study of lamotrigine in the treatment of affective instability in borderline personality disorder. Int Clin Psychopharmacol.

[11] First MB, Spitzer RL, Gibbon M, Williams JB, Benjamin L (1997). Structured Clinical Interview for DSM-IV Axis II Personality Disorders (SCID-II), version 2.0.

[12] Maffei C, Fossati A, Agostoni I, Barraco A, Bagnato M, Deborah D (1997). Interrater reliability and internal consistency of the structured clinical interview for DSM-IV axis II personality disorders (SCID-II), version 2.0. J Pers Disord.

[13] Tyrer P, Johnson T (1996). Establishing the severity of personality disorder. Am J Psychiatry.

[14] First MB, Spitzer RL, Gibbon M, Williams JB (2002). Structured Clinical Interview for DSM-IV-TR Axis I Disorders, Research Version.

[15] Gamma A, Angst J, Azorin JM, Bowden CL, Perugi G, Vieta E (2013). Transcultural validity of the Hypomania Checklist-32 (HCL-32) in patients with major depressive episodes. Bipolar Disord.

[16] Zanarini MC, Parachini EA, Boulanger JL, Frankenburg FR, Hennen J (2003). Zanarini Rating Scale for Borderline Personality Disorder (ZAN-BPD): a continuous measure of DSM-IV borderline psychopathology. J Pers Disord.

[17] Blum N, Pfohl B, John DS, Monahan P, Black DW (2002). STEPPS: a cognitive-behavioral systems-based group treatment for outpatients with borderline personality disorder--a preliminary report. Compr Psychiatry.

[18] Zanarini MC, Schulz SC, Detke HC, Tanaka Y, Zhao F, Lin D (2011). A dose comparison of olanzapine for the treatment of borderline personality disorder: a 12-week randomized, double-blind, placebo-controlled study. J Clin Psychiatry.

[19] Beck A, Ward C, Mendekson M, Mock JJE (1961). An inventory for measuring depression. Arch Gen Psychiatry.

[20] Beck AT, Steer RA, Garbin MG (1988). Psychometric properties of the Beck Depression Inventory: twenty-five years later. Clin Psychol Rev.

[21] Davidson KM (2007). Cognitive Therapy for Personality Disorders: A Guide for Clinicians.

[22] Palmer S, Davidson K, Tyrer P, Gumley A, Tata P, Norrie J (2006). The cost-effectiveness of cognitive behavior therapy for borderline personality disorder: results from the BOSCOT trial. J Pers Disord.

[23] Tyrer P, Nur U, Crawford M, Karlsen S, McLean C, Rao B (2005). The Social Functioning Questionnaire: a rapid and robust measure of perceived functioning. Int J Soc Psychiatry.

[24] Brooks R (1996). EuroQol: the current state of play. Health Policy.

[25] van Asselt AD, Dirksen CD, Arntz A, Giesen-Bloo JH, Severens JL (2009). The EQ-5D: a useful quality of life measure in borderline personality disorder?. Eur Psychiatry.

[26] British National Formulary (2013). British National Formulary 68.

[27] WHO ASSIST Working Group (2002). The Alcohol, Smoking and Substance Involvement Screening Test (ASSIST): development, reliability and feasibility. Addiction.

[28] Newcombe DA, Humeniuk RE, Ali R (2005). Validation of the World Health Organization Alcohol, Smoking and Substance Involvement Screening Test (ASSIST): report of results from the Australian site. Drug Alcohol Rev.

[29] Borschmann R, Barrett B, Hellier JM, Byford S, Henderson C, Rose D (2013). Joint crisis plans for people with borderline personality disorder: feasibility and outcomes in a randomized controlled trial. Br J Psychiatry.

[30] George CF, Peveler RC, Heiliger S, Thompson C (2000). Compliance with tricyclic antidepressants: the value of four different methods of assessment. Br J Clin Pharmacol.

[31] National Institute for Health and Care Excellence (2008). Guide to the Methods of Technology Appraisal.

[32] Curtis L (2014). Unit Costs of Health and Social Care 2013.

[33] Department of Health (2014). National Schedule of Reference Costs 2013–14 for NHS Trusts and NHS Foundation Trusts.

[34] Efron B, Tibshirani RJ (1993). An Introduction to the Bootstrap.

[35] Barber JA, Thompson SG (2000). Analysis of cost data in randomized trials: an application of the non-parametric bootstrap. Stat Med.

[36] Van Hout BA, Al MJ, Gordon GS, Rutten FH (1994). Costs, effects and cost-effectiveness ratios alongside a clinical trial. Health Econ.

[37] Fenwick E, Byford S (2005). A guide to cost-effectiveness acceptability curves. Br J Psychiatry.

[38] National Institute for Health and Care Excellence (2009). Borderline Personality Disorder – The NICE Guideline on Treatment and Management.

[39] National Health and Medical Research Council (2012). Clinical Practice Guideline for the Management of Borderline Personality Disorder.

[40] American Psychiatric Association Practice Guidelines (2001). Practice guideline for the treatment of patients with borderline personality disorder. Am J Psychiatry.

[41] Lieb K, Vollm B, Rucker G, Timmer A, Stoffers JM (2010). Pharmacotherapy for borderline personality disorder: Cochrane systematic review of randomized trials. Br J Psychiatry.

[42] Crawford MJ, MacLaren T, Reilly JG (2014). Are mood stabilisers helpful in treatment of borderline personality disorder?. BMJ.

